# Dyspnea in Chronic Fatigue Syndrome (CFS): Comparison of Two Prospective Cross-Sectional Studies

**DOI:** 10.5539/gjhs.v5n2p94

**Published:** 2012-12-12

**Authors:** Murugan K. Ravindran, Oluwatoyin Adewuyi, Yin Zheng, Rakib U. Rayhan, Uyenphuong Le, Christian R. Timbol, Samantha Merck, Rania Esteitie, Michelle Cooney, Charles Read, James N. Baraniuk

**Affiliations:** 1 Division of Rheumatology, Immunology and Allergy, Georgetown University, Washington DC, USA; 2 Division of Pulmonary, Critical Care and Sleep Medicine, Georgetown University, Washington DC, USA

**Keywords:** Medical Research Council Dyspnea Score, fatigue, CFS Severity Score, fibromyalgia, pulmonary function tests, total lung capacity, central sensitization

## Abstract

Chronic Fatigue Syndrome (CFS) subjects have many systemic complaints including shortness of breath. Dyspnea was compared in two CFS and control cohorts to characterize pathophysiology. Cohort 1 of 257 CFS and 456 control subjects were compared using the Medical Research Council chronic Dyspnea Scale (MRC Score; range 0-5). Cohort 2 of 106 CFS and 90 controls answered a Dyspnea Severity Score (range 0-20) adapted from the MRC Score. Subsets of both cohorts completed CFS Severity Scores, fatigue, and other questionnaires. A subset had pulmonary function and total lung capacity measurements. Results show MRC Scores were equivalent between sexes in Cohort 1 CFS (1.92 [1.72-2.16]; mean [95% C.I.]) and controls (0.31 [0.23-0.39]; p<0.0001). Receiver-operator curves identified 2 as the threshold for positive MRC Scores in Cohort 1. This indicated 54% of CFS, but only 3% of controls, had significant dyspnea. In Cohort 2, Dyspnea Score threshold of 4 indicated shortness of breath in 67% of CFS and 23% of controls. Cohort 2 Dyspnea Scores were higher for CFS (7.80 [6.60-9.00]) than controls (2.40 [1.60-3.20]; p<0.0001). CFS had significantly worse fatigue and other complaints compared to controls. Pulmonary function was normal in CFS, but Borg scores and sensations of chest pain and dizziness were significantly greater during testing than controls. General linear model of Cohort 2 CFS responses linked Dyspnea with rapid heart rate, chest pain and dizziness. In conclusion, sensory hypersensitivity without airflow limitation contributed to dyspnea in CFS. Correlates of dyspnea in controls were distinct from CFS suggesting different mechanisms.

## 1. Introduction

Dyspnea is a complex neural perception of shortness of breath, an inability to fully inflate or deflate the chest, and increased work of breathing (American Thoracic Society [ATS], 1999; Weinberger & Abu-Hasan, 2009). Perceptions of dyspnea and diffuse thoracic and throat discomfort are common in Chronic Fatigue Syndrome (CFS) (Fukuda et al., 1994), fibromyalgia (FM) (Wolfe et al., 1990; Wolfe et al., 2010), Gulf War Illness (GWI) (Veterans Administration [VA], 2008), Chronic Multisymptom Illness (Fukuda et al.,1998), and other related, overlapping symptom complexes. These syndromes share autonomic dysfunction (Burton, Rahman, Kadota, Lloyd, & Vollmer-Conna, 2010; Gur & Oktayoglu, 2008) and nociceptive central sensitization (Meeus & Nijs, 2007) that may contribute to pathophysiological mechanisms of dyspnea (Natelson et al., 2007; Caidahl et al.,1989; Lurie et al., 1990).

Alterations in neural mechanisms were anticipated given the neurological non-allergic rhinitis of CFS (Baraniuk et al., 2005), and intricate reflexes that connect nociceptive afferents from the nasal, laryngeal, bronchial and alveolar airways, and the chest wall. Processing of these afferents in the brain leads to efferent parasympathetic, sympathetic and motor outputs (Chen & Eldridge, 1997; Adam, 1998). Perceptions of dyspnea may involve imbalances between the afferent input from each of these different organs and neurological sensing systems (O’Donnell et al., 2007). The systemic nature of CFS complaints opens the possibility that dyspnea severity may be correlated with musculoskeletal pain, cardiothoracic and other complaints, quality of life, functional health and perceptions of well-being.

These postulated relationships were examined in two cohorts of subjects. Cohort 1 had 257 CFS and 456 control subjects. Modifications in the test evaluations were then introduced for the subsequent cohort.

Predictions were made based on prior knowledge from chronic obstructive lung disease, hyperventilation syndromes, and control subjects (ATS, 1999; Weinberger & Abu-Hasan, 2009; O’Donnell et al., 2007). We predicted that CFS subjects would have abnormalities on (a) spirometry such as vocal cord dysfunction, (b) provocation by hyperventilation (minute ventilation volume; MVV) with reversible airflow obstruction; (c) hyperinflation as seen in chronic obstructive pulmonary diseases (Weinberger & Abu-Hasan, 2009), and (d) significant correlations between dyspnea and anxiety measures.

## 2. Materials and Methods

### 2.1 Subjects

Cohort 1 included 257 CFS and 456 healthy control (HC) subjects from several prospective studies that used identical entry criteria and study instruments. Subjects reported their shortness of breath symptoms at various activity levels in nominal fashion (present vs. absent) using the Medical Research Council chronic dyspnea scale (MRC Score) (Manali et al., 2010; Paladini et al., 2010). The MRC Score ranged from 0 to 5 and provided point prevalences for shortness of breath induced by each activity. Univariate analysis of additional Cohort 1 data was designed to identify potential correlates of dyspnea.

After Cohort 1 was fully recruited, but before their data was analyzed, Cohort 2 of 106 CFS and 90 HC subjects were recruited to a single protocol. The scoring system was modified so Cohort 2 subjects reported the severity of each MRC query using 0 to 4 point anchored ordinal scales (*Section 2.2*). The sum was the Dyspnea Severity Score (Dyspnea Score; range 0 to 20).

History, physical examination, and screening blood work were used to classify subjects as CFS or HC, and to exclude those with HIV, pulmonary, congestive heart failure, cancer, autoimmune, and other chronic inflammatory illnesses that may have caused fatigue or shortness of breath. The HC and CFS subjects in both cohorts had similar sedentary lifestyles. This was reflected by lower than optimal scores for controls on many of the quality of life and other instruments. Because CFS was compared to sedentary controls, this study did not assess the full spectrum of dyspnea prevalence or severity across the entire general population. All subjects gave informed consent to participate in protocols approved by the Institutional Review Board (IRB) at Georgetown University. Subjects completed the protocol at Georgetown University, 3800 Reservoir Road, Washington, D.C., U.S.A.

### 2.2 CFS Case Designation Criteria

History and physical examination were used to identify CFS subjects. CFS requires nominal identification of severe fatigue lasting at least 6 months with no known precipitating cause plus at least 4 of the following 8 symptoms: (i) exhaustion in response to minor increases in exercise levels (“exertional exhaustion”); (ii) problems with cognition or short term memory; (iii) sleep disturbances; and discomfort affecting the (iv) throat, (v) cervical, axillary or inguinal lymph node regions, (vi) muscles, (vii) joints and (viii) headache (Fukuda et al., 1994). Subjects who did not meet these criteria were considered healthy control participants (HC). These nominal CFS criteria were augmented by self-reported symptom severity scores. Subjects scored the severity of each CFS criterion on a scale of none (score = 0), trivial (1), mild (2), moderate (3) or severe (4). This scaling was adapted from Wasserfallen et al. (1997). Inclusion of “trivial” allowed subjects to acknowledge slight complaints that did not significantly interfere with their activities of daily living or cause them to seek medical attention. A putative “extremely severe” category was found to be redundant by Cronbach's α tests and was merged with “severe”. The maximum sum of all 9 criteria was 36. The severity scores were used to compare CFS with HC responses, but not for CFS diagnosis. Fatigue was confirmed using the Multidimensional Fatigue Inventory (MFI) (Smets et al., 1995). Disability and poor quality of life were verified with the SF-36 (Ware & Sherbourne, 1995; Ware et al., 1994).

### 2.3 Dyspnea Questionnaires

Cohort 1 scored the Medical Research Council Chronic Dyspnea Scale (MRC Score) (Manali et al., 2010; Paladini et al., 2010). Subjects answered nominal (Yes/No; 1/0) questions about whether they developed shortness of breath: (1) at any time; (2) hurrying on level ground or walking up a slight hill; (3) walking on level ground with people of the same age; (4) walking at one's own pace on level ground; and (5) while washing or dressing Affirmative answers were scored 1 giving a range of 0 to 5. The MRC Score was the sum of all positive queries, and so was essentially a measure of the point prevalences of combinations of dyspnea complaints.

The MRC Score was expanded to the Dyspnea Severity Score (Dyspnea Score) to assess symptom severity in Cohort 2. The same 5 shortness of breath queries were each scored using the 0 to 4 point anchored ordinal scale (*Section 2.2*). Scores ranged from 0 to 20. Cohort 2 data were compared to the Borg Breathlessness Score and University of California, San Diego Shortness of Breath Questionnaire (Borg, 1970; Ries, 2005; Eakin et al., 1998).

### 2.4 Other Questionnaires

Cohort 1 subjects completed a Chronic Multiple Symptom Illness questionnaire by indicating in nominal fashion if they had experienced any of 42 symptoms for a 3 month period in the past year (Baraniuk et al., 1998). In Cohort 2, the severity of each symptom was assessed using the 0 to 4 point anchored ordinal scale. The symptoms covered neurocognitive, aural, respiratory, cardiac, musculoskeletal, gastrointestinal, urinary, and gynecological systems. Questions about Irritable Bowel Syndrome (Rome 1) (Lea, Hopkins, Hastleton, Houghton, & Whorwell, 2004) were not included in this statistical analysis, but will be reported elsewhere. Pain qualities were scored with the McGill short form Pain Score with its Affective and Sensory subscales (Melzack, 1987). Relative activity levels were compared using the Minnesota Heart Survey (Folsom et al., 1985). Affective and anxiety problems were assessed using the Beck Depression Inventory (Beck, Steer, Ball, & Ranieri, 1996), Center for Epidemiology – Depression score (CES-D) (Geisser, Roth, & Robinson, 1997; Radkiff, 1977), seven question Generalized Anxiety Diagnosis Screener (GAD-7) (Lowe et al., 2008), and Speilberger's State – Trait Anxiety Inventory (STAI-Y1) (Okun, Stein, Bauman, & Silver, 1996).

### 2.5 Fibromyalgia and Systemic Hyperalgesia

Fibromyalgia (FM) was assessed using the 1990 American College of Rheumatology criteria (Wolfe et al., 1990). Diffuse, widespread pain affecting all 4 quadrants and the axial skeleton plus tenderness to thumb pressure at ≥ 11 of 18 traditional tender points were required. The 2010 FM criteria (Wolfe et al., 2010) were not used since data collection began before their publication.

### 2.6 Spirometry in Cohort 2

Subjects performed three flow – volume loops to assess intra – and extra – thoracic obstruction while standing using a Spirometrix 2500 LTE Spirometer (Bayview Respiratory, Baltimore, MD) with disposable mouth pieces, Composite standards, and 3L volume calibration for each subject. Subjects immediately scored their sensation of shortness of breath with the Borg Scale (Borg, 1970), and the “intensity” and “tolerability” of their most extreme respiratory effort on an adapted 20 point anchored ordinal pain scale (Gracely & Dubner, 1987). Subjects were noted to develop dizziness or lightheadedness, and so the same 20 point anchored ordinal scale was adopted to assess this sensation as well. Next, subjects performed a minute ventilation volume (MVV) maneuver. They scored their Borg, intensity, tolerability and dizziness scores immediately after the MVV. Three post – MVV flow – volume loops were performed to determine if the hyperventilation induced any bronchospasm or narrowing of the extrathoracic airway. Sensations were measured for a third time. The % predicted of each spirometry variable was compared between CFS and HC subjects.

### 2.7 Whole Body Plethysmography

Total lung capacities (TLC) were measured in a Plexiglas plethysmograph (Vmax Encore System, VIASYS Health Care Respiratory Care, Inc.). Calibration, quality control, and standards followed established American Thoracic Society and European Respiratory Society guidelines (Wanger et al., 2005).

### 2.8 Statistics

Individual symptom scores and questionnaire Domain Scores were described by their mean and 95% confidence interval (95% C.I.) (Gardner & Altman, 1989) calculated using SAS 9.2 (SAS Institute, Cary, NC). Significant differences between nominal, dichotomous results of CFS and HC groups from both Cohorts were determined by Fisher's Exact Test with p < 0.05 for significance. Continuous and ordinal data were compared by 2-tailed unpaired Student's t-test (Swinscow & Campbell, 2002). All probabilities were corrected for multiple comparisons (n=148) using the Bonferroni method. Pearson's correlations were used to identify pairs of variables that were highly associated with each other. The explained variances (R2) between the MRC Scores of Cohort 1 and Dyspnea Scores of Cohort 2 were calculated for each variable. Preliminary calculations showed that results from male and female subsets of each CFS and HC group were equivalent within each Cohort. Correlation coefficients for the CFS and HC groups were relatively low indicating “ceiling” and “floor” effects for the distributions of scores. Frequency distributions were plotted for MRC and Dyspnea Scores to determine the mean and 95^th^ percentiles; the latter were used to set thresholds for positive Scores. Receiver – operator curves identified the optimum sensitivity and specificity for each scoring system. Generalized linear models with backwards elimination were used to define the variables that were most significantly related to Dyspnea Scores in the Cohort 2 CFS and HC groups.

## 3. Results

### 3.1 Demographics of Cohort 1

Gender and ages were not significantly different between the CFS and HC groups within each cohort after Bonferroni corrections (Table 1). Cohort 1 was younger than Cohort 2. Shortness of breath was present in 56% of CFS and 12% of HC in Cohort 1. The queries accounted for 0.63 of the expected variance in the MRC Score. CFS had significantly higher MRC Scores (1.92 [1.72-2.16]) than HC (0.31 [0.23-0.39]; p < 0.0001 by Bonferroni corrected t-test). The frequency distribution for MRC Scores (Figure 1) showed that over 80% of HC had scores of zero. The 95^th^ percentile was 2 on the 5 point scale. MRC Scores were zero for 35% of CFS subjects, then between 10% and 20% for each score of 1 and higher. The Cohort 1 receiver – operator curve indicated high specificities but low sensitivities for all scores. Optimum sensitivity (0.65) and specificity (0.90) were found with a MRC Score of 1 (grey square to the left of the dashed diagonal line). When the 95^th^ percentile for the HC group was used, a score of 2 (star symbol) gave sensitivity of 54% and specificity of 97%. Positive MRC Scores were defined as 2 and greater.

**Table 1:** Nominal MRC shortness of breath scores (MRC Score) and prevalence of each query in the Cohort 1 CFS, HC, female, and male subgroups (mean [95% C.I.])

Cohort 1	CFS			HC		

	Females	Males	All CFS	Females	Males	All HC
N (% of group)	213 (83%)	44 (17%)	257	330 (72%)	126 (28%)	456

Age	44.2 [42.8-45.7]	41.8 [38.9-44.6]	43.6 [42.2-44.7]	41.3 [39.8-42.8]	39.8 [37.4-42.3]	41.0 [39.6-42.2]
SOB at any time *	57%	55%	56%	14%	7%	12%
SOB while hurrying *	62%	50%	60%	15%	8%	13%
SOB walking with others *	42%	34%	40%	4%	3%	4%
SOB walking at your own pace *	25%	23%	25%	2%	2%	2%
SOB washing or dressing *	23%	20%	23%	1%	2%	1%
MRC Score (0-5) **	2.00 [1.76-2.24]	1.66 [1.09-2.23]	1.92 [1.72-2.16]	0.35 [0.25-0.44]	0.22 [0.08-0.36]	0.31 [0.23-0.39]

* p < 0.0001 when All CFS and All HC responses were compared by Fisher’s Exact Tests (bold)

** p < 0.0001 by 2-tailed unpaired Student’s t-test with Bonferroni correction (bold)

**Figure 1:**
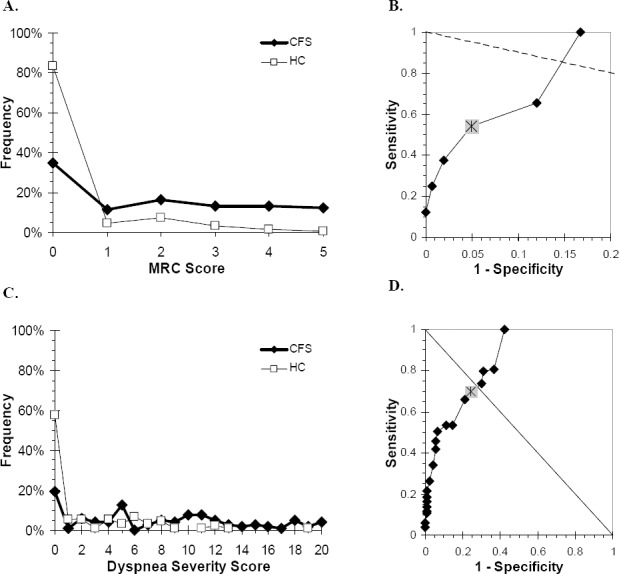
A. Frequency distribution of MRC Scores in Cohort 1. B. Receiver – operator curve for MRC Scores in Cohort 1 CFS and HC subjects. C. Frequency distribution of MRC Scores in Cohort 2. D. The Cohort 2 Dyspnea Score receiver – operator curve determined that a positive threshold score of 4 (star to the left of the diagonal line) had a sensitivity of 67% and specificity of 77%

### 3.2 Demographics of Cohort 2

Females made up 62% of the CFS and 44% of the HC groups (Table 2). Ages were equivalent. MRC Scores were not collected for Cohort 2. Dyspnea Scores for CFS (7.8 [6.6-9.0]) were significantly higher than for HC (2.4 [1.6-3.2]; p < 0.0001 by t-test). Scores for all individual queries were significantly different between CFS and HC (p < 0.0001).

**Table 2:** Cohort 2 Dyspnea Severity Scores. Scores for each questionnaire item (0 to 4), their sum (Dyspnea Score), Borg and UCSD shortness of breath questionnaire results were compared between the CFS and HC groups (mean [95% C.I.]). Scores for individual items were correlated with the Dyspnea Severity Score to determine the explained variances (Pearson’s R^2^)

Cohort 2	CFS			HC		

	Females	Males	All CFS	Females	Males	All HC
N (% of group)	67 (62%)	41 (38%)	CFS = 106	39 (44%)	51 (57%)	HC = 90

Age	51.2 [48.4-54.0]	49.5 [46.4-52.6]	50.0 [47.1-52.9]	47.3 [42.6-52.1]	52.1 [48.5-55.7]	50.6 [48.5-52.6]
SOB at any time *	1.62 [1.28-1.95]	1.87 [1.41-2.33]	1.74 [1.47-2.01]	0.51 [0.27-0.77]	0.43 [0.19-0.67]	0.47 [0.29-0.64]
SOB while hurrying *	2.08 [1.72-2.43]	2.03 [1.56-2.49]	2.12 [1.84-2.40]	0.92 [0.56-1.28]	0.51 [0.24-0.77]	0.70 [0.48-0.91]
SOB walking with others *	1.61 [1.24-1.98]	1.76 [1.30-2.22]	1.70 [1.41-1.99]	0.82 [0.45-1.19]	0.39 [0.17-0.62]	0.58 [0.37-0.78]
SOB walking at own pace *	1.28 [0.93-1.63]	1.39 [0.99-1.80]	1.33 [1.07-1.60]	0.59 [0.25-0.93]	0.29 [0.10-0.49]	0.42 [0.24-0.61]
SOB washing or dressing *	0.98 [0.67-1.30]	1.11 [0.70-1.52]	1.06 [0.81-1.31]	0.21 [0.02-0.39]	0.23 [0.02-0.45]	0.22 [0.08-0.37]
Dyspnea Severity Score (0-20) *	7.54 [6.00-9.07]	8.16 [6.16-10.15]	7.80 [6.60-9.00]	3.15 [1.86-4.44]	1.86 [0.84-2.87]	2.40 [1.6 -3.2]
Borg Score (0-10) *	1.63 [1.22-2.05]	1.93 [1.33-2.54]	1.7 [1.4-2.1]	0.54 [0.24-0.84]	0.45 [0.20-0.69]	0.5 [0.3-0.7]
UCSD Dyspnea Score (0-120) *	33.4 [26.8-40.0]	29.7 [20.3-39.1]	32.1 [26.7-37.4]	14.0 [8.3-19.7]	8.27 [5.1-11.5]	10.7 [7.6-13.7]

* p < 0.0001 between All CFS and All HC values (columns in bold) by 2-tailed unpaired Student’s t-tests after Bonferroni corrections

Severities for each symptom were trivial to mild for CFS, and absent to trivial for HC. Explained variances between Dyspnea Score and each of the queries ranged from 0.65 to 0.82 for CFS, and 0.48 (washing or dressing) to 0.86 for HC. Sensitivity was 67% and specificity 77% by receiver – operator analysis when a score of 4 out of 20 defined a positive test (Figure 1). Borg and UCSD Dyspnea Scores were significantly higher in Cohort 2 CFS than HC (p < 0.0001) (Table 2). Dyspnea Scores correlated with both the Borg (R2 = 0.50) and UCSD (R2 = 0.56) results in CFS and HC (0.39 and 0.59, respectively).

### 3.3 CFS Severity Score

The severity score for each CFS case designation criterion was significantly higher for CFS than HC subjects in both Cohorts 1 and 2 (p < 0.0001 for each comparison) (Table 3). Only Exertional Exhaustion was consistently correlated with MRC and Dyspnea Severity Scores in the 2 cohorts (range for R2 of 0.10 to 0.21). The absence of correlations between shortness of breath and the other CFS criteria suggested that mechanisms of dyspnea may be different from those responsible for CFS pain and cognitive complaints.

**Table 3:** Cohort 1 and 2 CFS Severity Scores. The severity of each CFS criterion (mean [95% C.I.]) was compared between CFS and HC groups. Explained variances (R^2^) for correlations with MRC Scores (Cohort 1) and Dyspnea Severity Scores (Cohort 2) were shown. All CFS scores were significantly higher than HC in the two Cohorts

Cohorts	CFS Criterion Severity Scores (mean [95% C.I.])		R^2^	

			MRC Score	
**Cohort 1**	CFS (n = 127) *	**HC (n = 186)**	**CFS**	**HC**
Fatigue	3.52 [3.43-3.61]	0.78 [0.63-0.93]	0.04	0.04
Cognition	2.75 [2.55-2.94]	0.69 [0.53-0.86]	0.05	0.07
Sore throat	1.53 [1.31-1.74]	0.37 [0.25-0.49]	0.02	0.02
Sore lymph nodes	1.23 [1.00-1.46]	0.24 [0.13-0.35]	0.01	0.08
Myalgia	3.12 [2.92-3.31]	0.59 [0.45-0.74]	0.01	0.10
Arthralgia	2.47 [2.22-2.72]	0.48 [0.34-0.62]	0.01	0.07
Headache	2.61 [2.41-2.82]	0.82 [0.66-0.99]	0.003	0.08
Sleep disturbances	3.25 [3.10-3.42]	0.91 [0.73-1.10]	0.001	0.08
Exertional exhaustion	2.92 [2.71-3.14]	0.41 [0.27-0.55]	0.11	0.10

			**Dyspnea Score**	
**Cohort 2**	CFS (n = 106) *	**HC (n =90)**	**CFS**	**HC**
Fatigue	3.60 [3.51-3.70]	1.13 [0.88-1.39]	0.06	0.17
Cognition	2.88 [2.69-3.06]	0.81 [0.59-1.04]	0.004	0.15
Sore throat	1.34 [1.11 -1.57]	0.23 [0.10-0.36]	0.03	0.02
Sore lymph nodes	1.27 [1.02-1.53]	0.11 [0.02-0.20]	0.11	0.06
Myalgia	3.11 [2.91-3.32]	1.04 [0.76-1.31]	0.08	0.05
Arthralgia	2.92 [2.69 -3.14]	1.06 [0.80-1.31]	0.08	0.06
Headache	2.54 [2.30 -2.78]	0.72 [0.49-0.95]	0.007	0.04
Sleep disturbances	3.46 [3.30-3.62]	1.24 [0.96-1.52]	0.007	0.02
Exertional exhaustion	3.32 [3.13 -3.51]	0.96 [0.70-1.23]	0.21	0.21

* p < 0.0001 by 2-tailed unpaired Student’s t-tests followed by Bonferroni corrections

### 3.4 Fatigue Ratings

Fatigue was verified for both cohorts by higher Multidimensional Fatigue Inventory domain scores compared to HC (Table 4). Fatigue Domains of CFS groups did not correlate with MRC (Cohort 1) or Dyspnea Severity Scores (Cohort 2). In contrast, the Cohort 2 HC group had correlations of Dyspnea Scores with each domain except Mental Fatigue. This suggested that the mechanisms responsible for perceptions of fatigue and shortness of breath in HC may not be applicable to CFS subjects.

**Table 4:** Verification of increased fatigue in CFS. Each Multidimensional Fatigue Inventory (MFI) Domain score was significantly higher in CFS than HC for both cohorts.. Domain scores were correlated with MRC Score (Cohort 1) Dyspnea Score (Cohort 2) and explained variances (Pearson’s R^2^) assessed.

MFI Domains for Cohorts 1 and 2	Domain Scores Mean [95% C.I.]		Explained Variance (R^2^)	
	**CFS**	**HC**	**CFS**	HC
**Cohort 1 (n)**	**N = 147**	**n = 245**	**MRC Score**	
General Fatigue **	16.4 [15.8-16.9]	10.2 [9.6-10.7]	0.08	0.02
Physical Fatigue **	14.6 [13.9-15.2]	9.1 [8.6-9.7]	0.08	0.09
Mental Fatigue **	13.6 [12.9-14.3]	8.8 [8.3-9.3]	0.08	0.03
Reduced Activity **	12.8 [12.40-13.5]	8.1 [7.7-8.6]	0.05	0.05
Reduced Motivation **	11.1 [10.5-11.8]	7.8 [7.1-8.4]	0.05	0.03

**Cohort 2**	**N = 104**	**n = 87**	**Dyspnea Score**	
General Fatigue **	15.3 [14.6-16.1]	11.4 [10.6-12.3]	0.0004	0.18
Physical Fatigue **	13.7 [13.1-14.4]	11.2 [10.2- 12.2]	0.0003	0.29
Mental Fatigue **	12.4 [11.6-13.1]	9.5 [8.6- 10.4]	0.0001	0.03
Reduced Activity **	13.7 [13.0-14.4]	10.3 [9.3-11.2]	0.0007	0.16
Reduced Motivation *	11.3 [10.6-12.0]	10.1 [9.3-10.9]	0.001	0.15

* p = 0.03

** p < 0.0001 between CFS and HC results by Bonferroni corrected 2-tailed unpaired Student’s t-tests

### 3.5 Quality of Life in CFS

SF-36 indicated quality of life and disability were worse in CFS than HC. CFS subjects had SF-36 scores below 50 for all domains except Role-Emotional and Mental Health (Table 5). Scores for HC groups in both cohorts were ≥ 59 with the exception of Vitality in Cohort 1 (48.5). This indicated the HC groups had important limitations compared to exceptionally healthy subjects who would be expected to have scores ≥ 80. MRC Scores did not correlate with SF-36 domains except for Physical Functioning in CFS (R2 = 0.20). The wider range of Dyspnea Scores compared to MRC Scores generated higher correlations for Physical Functioning in CFS (R2 = 0.31) and HC (R2 = 0.44); and General Health (R2 = 0.27) and Social Functioning (R2 = 0.23) in HC. The relatively low SF-36 scores for HC subjects suggested that generally poor quality of life and factors such as age – related declines in fitness were not responsible for the CFS – related dyspnea.

**Table 5:** Verification of reduced quality of life in CFS using SF-36

SF-36 Domains	Domain Scores Mean [95% C.I.]		Variance (R^2^)	

	CFS	HC	CFS	HC
**Cohort 1**	**n = 138**	**n = 240**	**SOB Score**	
Physical Functioning **	49.0 [44.2-53.8]	71.8 [67.5-76.1]	0.20	0.06
Role-Physical **	17.9 [12.3-23.5]	59.1 [53.3-64.8]	0.06	0.07
Vitality **	25.0 [21.6-28.4]	48.5 [45.0-52.1]	0.04	0.07
Bodily Pain **	35.6 [31.6-39.7]	59.0 [54.2-63.8]	0.06	0.03
General Health **	38.5 [34.0-43.1]	65.7 [61.0-70.4]	0.04	0.05
Social Functioning *	45.6 [40.6-50.5]	64.1 [59.1-69.0]	0.07	0.05
Role-Emotional	50. 5 [42.6-58.4]	67.8 [57.2-78.4]	0.006	0.02
Mental Health	57.2 [53.4-61.1]	64.6 [60.1-69.1]	0.006	0.06

**Cohort 2**	**n = 102**	**n = 87**	**Dyspnea Score**	
Physical Functioning **	41.8 [36.9-46.8]	74.0 [67.5-80.4]	0.31	0.44
Role-Physical **	13.7 [8.3-19.2]	63.8 [54.4-73.2]	0.04	0.18
Vitality **	18.9 [15.7-22.0]	56.0 [51.1-61.0]	0.06	0.17
Bodily Pain **	31.6 [27.8-35.4]	71.6 [66.0-77.2]	0.09	0.16
General Health **	35.4 [31.6-39.1]	61.5 [57.1-65.8]	0.15	0.27
Social Functioning **	33.3 [28.5-38.2]	80.3 [75.0-85.6]	0.10	0.23
Role-Emotional	55.9 [47.1-64.7]	84.1 [77.4-90.8]	0.000002	0.09
Mental Health **	60.6 [56.3-64.8]	76.0 [72.6-79.3]	0.008	0.11

* p = 0.0006

** p < 0.0001 for differences between CFS and HC groups by 2-tailed unpaired Student’s t-tests with Bonferroni corrections

### 3.6 Minnesota Survey and McGill Pain Short Form

The CFS and HC groups of both cohorts had similar sedentary lifestyles based on the Minnesota Heart Survey (Table 6). The McGill Short Form Pain Affective subscale was significantly higher in both CFS cohorts compared to HC. CFS subjects of Cohort 2 gave the most pain descriptors (Sensory subscale). The McGill scores were well correlated with MRC Scores in CFS subjects of Cohort 1 (R2 = 0.22 to 0.24), but more weakly with Dyspnea Scores in Cohort 2 (R2 = 0.09 to 0.13). Dyspnea may have been perceived as an interoceptive equivalent to pain by CFS subjects.

**Table 6:** Activity and pain measures. Differences in these outcomes (mean [95% C.I.) and correlations with MRC and Dyspnea scores (Pearson’s R^2^) were evaluated for Cohort 1 and 2 CFS and HC groups

**Cohort 1**	**CFS**	**HC**	**MRC Score (R^2^)**	
Minnesota Heart Survey	n=77	n=255	CFS	HC
	10.0 [5.3-14.7]	15.5 [11.1-19.8]	0.05	0.002
McGill Pain Short Form	n=53	n=103		
Affective *	2.9 [2.2-3.5]	0.9 [0.5-1.2]	0.22	0.06
Sensory	8.7 [6.9-10.4]	6.0 [4.8-7.3]	0.24	0.01
Total	11.4 [9.1-13.7]	6.9 [5.3-8.4]	0.24	0.02

**Cohort 2**	**CFS**	**HC**	**Dyspnea Score (R^2^)**	
Minnesota Heart Survey	n=90	n=75	CFS	HC
	6.6 [3.1-10.1]	8.88 [6.1-11.7]	0.04	0.06
McGill Pain Short Form	n=103	n=89		
Affective *	4.5 [3.9-5.1]	1.29 [0.8-1.8]	0.08	0.09
Sensory *	15.5 [14.1-17.0]	5.21 [3.7-6.7]	0.14	0.12
Total *	20.0 [18.1-21.9]	6.51 [4.6-8.4]	0.14	0.12

* p < 0.0001 between CFS and HC after Bonferroni corrections of 2-tailed unpaired Student’s t-tests

### 3.7 General Linear Modeling of Dyspnea Scores

A general linear model of Cohort 2 outcomes identified symptoms that were significantly associated with Dyspnea Scores. In the CFS group, rapid heart rate (p < 0.0001), muscle spasms (p = 0.0006) and dizziness (p = 0.004) were identified (p < 0.0001 for model). This analysis in HC identified chest pain (p<0.0001), rapid heart rate (p = 0.004), burning urination (p = 0.016), fingers sensitive to the cold (p = 0.023), dry eyes (p = 0.024) and numbness (p = 0.027).

### 3.8 Psychometric Components

Psychometric measures of depressive affect (CES-D and BDI) and anxiety (STAI-Y1) were significantly higher in CFS than HC (p < 0.001) of Cohort 2 (Table 7). Female and male subjects had equivalent responses within each group. In contrast, GAD-7 scores had wide ranges in both CFS and HC groups so that there were no significant differences between CFS, HC, female or male subgroups. GAD-7 scores of 10 to 14 occur in 5% of the general population, with 1% having scores of 15 or higher (Lowe et al., 2008). CFS subjects had no significant relationships between Dyspnea Scores or gender with depression or anxiety test results (R2 ≤ 0.03). However, HC scores for CES-D and STAI-Y1 were correlated with Dyspnea Score (R2 = 0.29 and 0.20, respectively). Anxiety and affective complaints were not associated with shortness of breath in CFS.

**Table 7:** Cohort 2 Depression and Anxiety State Questionnaires. The mean [95% C.I.] results for CES-D, STAI-Y1 and BDI were significantly higher for CFS than HC groups. Only the HC group had significant correlations (R^2^) between these questionnaires and Dyspnea Score (bold cells)

Groups	CFS			HC		

	Female	Male	All CFS	Female	Male	All HC
CES-D *	20.7 [17.9-23.6] (n=65)	25.5 [21.5-29.5] (n=39)	22.5 [20.2-24.9] (n=104)	10.0 [7.2-12.8] (n=37)	9.4 [6.7-12.1] (n=51)	9.7 *** [7.7–11.6] (n=88)
STAI-Y1 *	41.6 [37.9-45.3] (n=61)	49.1 [43.9-54.4] (n=36)	44.4 [41.3–47.4] (n=97)	35.7 [31.8-39.6] (n=37)	32.5 [29.4-35.6] (n=50)	33.9 ** [31.4–36.3] (n=87)
BDI *	11.8 [9.8-13.8] (n=64)	17.9 [13.8-21.9] (n=37)	14.0 [12.0–16.0] (n=101)	8.3 [5.7-10.9] (n=39)	8.3 [5.9-10.7] (n=50)	8.3 [6.6–10.1] (n=89)
GAD-7	7.6 [5.6-9.7] (n=39)	5.9 [2.3-9.4] (n=14)	7.2 [5.4–8.9] (n=53)	4.2 [1.1-7.3] (n=17)	4.1 [1.9-6.4] (n=22)	4.2 [2.4–5.9] (n=39)

* p < 0.001 between All CFS and All HC scores by 2-tailed unpaired Student’s t-test after Bonferroni corrections

** HC had explained variances of 0.20 for STAI-Y1

*** 0.29 for CES-D with Dyspnea Scores

### 3.9 Assessment of Fibromyalgia

Fibromyalgia (1990 criteria) (Wolfe et al., 1990) and CFS were co-morbid conditions in 42% of Cohort 1. MRC Scores for CFS (2.3 [1.9-2.6]) and CFS/FM (1.7 [1.4-2.0]) subjects were not significantly different. HC had scores of 0.3 [0.2-0.4] (p < 0.0001 vs. both CFS subsets).

FM was present in 47% of Cohort 2 CFS subjects. Dyspnea Scores were equivalent for CFS/FM (9.0 [6.9-11.0]) and CFS (6.7 [4.8-8.5]) subgroups (p = 0.11). Both were significantly higher than HC (1.9 [0.9-3.0]) (p < 0.0006 vs. both CFS subsets). Pain thresholds by dolorimetry were significantly lower in CFS/FM (1.9 kg [1.5-2.3]) than CFS (3.7 kg [3.1-4.2]; p < 0.0001) and HC (4.9 kg [4.04-5.83]; p < 0.0001) groups. CFS and HC were not significantly different after Bonferroni correction.

### 3.10 Pulmonary Function testing, Spirometry, and Total Lung Capacity

Pulmonary function tests in Cohort 2. CFS (n=59) and HC (n=31) subjects had spirometry, minute ventilation volume (MVV), and post-MVV spirometry. CFS subjects had significantly higher Borg Scores following PFT's before and after the MVV maneuver (Figure 2). CFS subjects had higher complaints of dizziness after each component of the PFT testing. The higher dizziness after MVV may be related to complaints of lightheadedness in relation to dyspnea (Table 7). CFS subjects had significantly greater sensations of chest pain throughout the PFT testing (p ≤ 0.03). The wide 95% confidence intervals for each subjective measure in the CFS subjects suggested a broad range in the levels of complaints.

**Figure 2:**
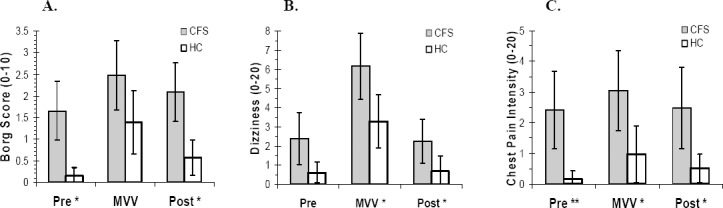
Symptoms associated with pulmonary function testing. A. Borg Scores were measured after the first set of PFT's (Pre), MVV, and final set of PFT's (Post) (mean; 95% C.I.; * p = 0.002 by 2-tailed unpaired Student's t-test between CFS and HC). B. Dizziness following each set of tests (0 to 20 scale; * p < 0.04). C. Chest pain intensities were significantly higher in CFS than control subjects (* p = 0.03; ** p = 0.008)

Spirometry was normal and equivalent for the CFS and HC groups both before and after MVV (Figure 3). However, the aggregate of all peak expiratory flow rates was significantly lower in CFS (391 L/min [266-416]) than HC subjects (436 L/min [403-469]; p = 0.037). Peak inspiratory flow rates were not different (CFS: 240 L/min [218-262]; HC 266 L/min [238-294]). The hyperventilation of the MVV did not induce any intra- or extra- thoracic airflow obstruction.

**Figure 3:**
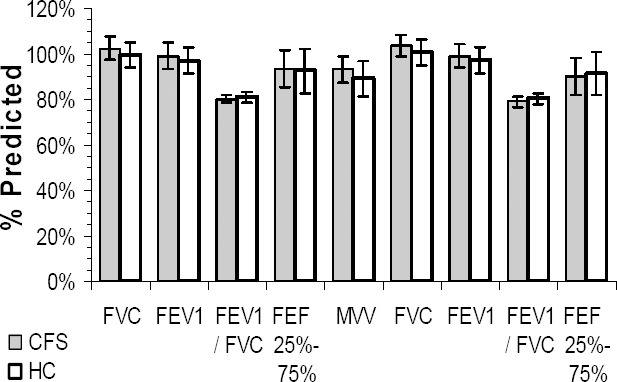
Pulmonary function test results before and after MVV for CFS (grey bars) and HC (white bars) (mean; 95% C.I.)

TLC was not significantly associated with body mass index (BMI) in 18 CFS subjects (Figure 4). TLC was decreased in one subject who was recuperating from a recent pneumonia, and one presumed CFS subjects who was later diagnosed with *Mycobacterium avium-intracellulare*. None of this subject's data were included in the statistical analyses. This TLC result was included as a reminder that other illnesses may be identified as the ultimate cause(s) of CFS complaints. Two CFS subjects (13%) had elevated TLC without explanation. Alpha-1-antitrypsin genotypes were normal (MM) (Blanco, Canto, De Serres, Fernandez-Bustillo, & Rodriguez, 2004). In general, hyperinflation was not a major factor in the dyspnea of CFS.

**Figure 4:**
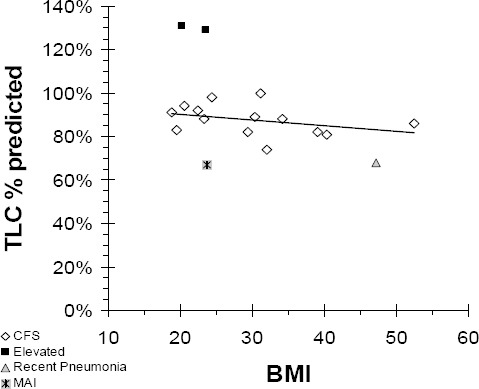
Total lung capacity (TLC). TLC was not significantly associated with body mass index (BMI). Outliers had elevated TLC (black squares), recent pneumonia (grey triangle), and *Mycobacterium avium-intracellulare* (MAI, cross)

## 4. Discussion

These findings suggest that the mechanism(s) of dyspnea in CFS may be different from other conditions. Dyspnea is a sensation that requires intact afferent and efferent neural pathways, and may represent an imbalance between competing sensory input systems that gauge the musculoskeletal work of breathing, and the respiratory consequences of those efforts. Nasal trigeminal afferent receptors monitor the passage of air by sensing evaporative cooling as air moves in and out of the nostrils. Temperature sensitive ion channels such as transient receptor potential ankyrin 1 (TRPA1) and menthol – sensitive TRP melanostatin 8 (TRPM8) on Aδ neurons may play roles in transducing the airway cooling into the perception of nasal patency (Baraniuk, 2010). This afferent input to brainstem centers may help determine the inspiratory muscle effort required to inhale each breath.

Proprioceptive stretch receptors in the lung, respiratory muscles and tendons may provide information about the extent of inspiratory effort and elastic recoil during exhalation (O’Donnell et al., 2007). Acute changes in arterial pH and pCO2 likely have rapid effects in the carotid body to add a chemical dimension to the calculation of required respiratory effort. Medullary respiratory motor centers coordinate the diaphragmatic and musculoskeletal contractions required to satisfy these afferent stimuli. An imbalance in any segment of this tightly regulated pathway may lead to a distressing urge to breathe that is independent of inspiratory muscular effort. Brain imaging studies indicate limbic system activation during dyspnea (Evans et al., 2002). This affective component may occur as the conscious perception of hypoventilation in acute and chronic bronchial and lung parenchymal diseases, or hyperventilation during exercise or anxiety (Weinberger & Abu-Hasan, 2009). In each situation, the increasing central neural motor output and inspiratory muscle effort becomes dissociated from the lagging afferent sensing of sufficient air intake, and generates a sensation of unsatisfied inspiration (O’Donnell & Lavaeneziana, 2007; Ofir, Laveneziana, Webb, Lam, & O’Donnell, 2008).

CFS reported more frequent and severe dyspnea than HC subjects based on the MRC Score, Dyspnea Score, Borg, and UCSD Shortness of Breath Questionnaire. Levels of dyspnea were absent to trivial in HC, but were skewed to higher severities in CFS. Exertional exhaustion, a key determinant of CFS, was correlated with MRC and Dyspnea Scores. However, neither fatigue scores nor MFI fatigue domains were associated with dyspnea. The Physical Functioning domain score from the SF-36 indicated more dyspnea – related disability in CFS than HC. MRC Scores were correlated with McGill Pain descriptors in Cohort 1 CFS subjects.

Co-morbid FM was found in 42% of Cohort 1 and 47% of Cohort 2 CFS subjects suggesting shared pathophysiological mechanisms. Both CFS and FM have been associated with dyspnea (Weiss et al., 1998). The threshold of 2 for the MRC Score in Cohort 1 (Figure 2) was the same as the threshold in 87 FM subjects (Caidahl et al., 1989). Significant dyspnea was identified in 31% of those FM subjects (Caidahl et al., 1989). Dyspnea Scores in our CFS subjects were correlated with rapid heart rate, muscle spasms and dizziness in the general linear model. Chest pain and other thoracic symptoms have been associated with FM (Pellgrino, 1990). FM was identified in 5% (Wise, Semble, & Dalton, 1992) to 30% (Murkerji, Mukerji, Alpert, & Selukar, 1995) of noncardiac chest pain cases. The thoracic complaints may be related to “small heart syndrome” as described in Japanese CFS subjects (Miwa & Fujita, 2008).

CFS subjects experienced significant perceptions of lightheadedness, chest pain and breathlessness during spirometry and hyperventilation (MVV). Normal spirometry (Figure 7) without bronchospasm or hyperinflation (Figure 8) has generally been found in CFS and FM groups. However, significantly lower peak expiratory flow rates were found in Cohort 2 CFS subjects that may be consistent with the reduced inspiratory and expiratory airway pressures reported for FM (Lurie et al., 1990). Baseline hyperventilation may lead to hypocapnia in CFS (Lavietes et al., 1996). A symptom complex of hypocapnia, dyspnea, and orthostatic hypotension was described in about 21% of CFS, but only 3% of healthy control subjects (Natelson et al., 2007; Cook, Nagelkirk, Poluri, Mores, & Natelson, 2006). Exercise to the anaerobic threshold has not been a physiological limitation for CFS subjects when compared to sedentary controls (Sargent et al., 2002). However, CFS subjects performed significantly worse than controls when two sets of exercise to anaerobic threshold were done 24 hr apart (Vermeulen et al., 2010). Complaints of pain with peak exercise were higher in CFS/FM subjects compared to CFS alone or sedentary controls suggesting that systemic hyperalgesia may play a confounding role in the perception of shortness of breath (Cook et al., 2006).

Anxiety was more severe in CFS based on STAI-Y1 and GAD-7 results (Table 7). Although STAI-Y1 was correlated with Dyspnea Score in HC, the CFS subjects had no correlations between anxiety and Dyspnea Scores. In contrast, anxious, but otherwise healthy subjects may generate lower peak airway pressures that lead to perceptions of dyspnea but without other pulmonary dysfunction (Tiller, Pain, & Biddle, 1987). Comparable results were found in COPD subjects with and without panic attacks (Livermore et al., 2008; Giardino et al., 2010). In asthma, the perception of dyspnea is worse with more severe inflammation, older age, depression (Foschino-Barbaro et al., 2010), and anxiety-trait (Nowobilski et al., 2007). These findings suggest that mechanism(s) of dyspnea in parenchymal lung diseases differ from CFS. The correlation of Dyspnea Scores with anxiety measures in HC but not CFS suggests that generalizations based on HC subjects may not be valid for CFS.

These data did not support the predictions made in the Introduction that were based on studies in chronic obstructive lung disease, hyperventilation syndromes, and other subjects (ATS, 1999; Weinberger & Abu-Hasan, 2009; O’Donnell et al., 2007). Spirometry did not identify reversible intra- or extrathoracic airflow obstruction. Methacholine or propranolol provocations may be needed to induce obstruction. Two CFS subjects had hyperventilation without alpha-1-antitrypsin deficiency. This suggests excessive lung parenchymal and chest wall stretch was not a major contributor to their dyspnea. However, peripheral sensory neuron sensitization may account for increased symptoms without measurable changes. This was supported by Figure 2. Tidal, static and forced volume testing may be needed to show differences from controls. Only HC subjects had correlations of dyspnea with affective and anxiety changes. Verification of previously reported hypocapnia at rest is necessary (Lavietes et al., 1996). Pulmonary stress tests for anaerobic threshold, muscular tolerance, deconditioning, and assessments of post-exertional exacerbations of fatigue and pain are likely to offer additional insights into dyspnea in CFS.

## 5. Conclusion

Two cohorts of CFS subjects had significantly greater complaints of dyspnea, systemic pain, other symptoms, and disability compared to healthy controls. Measures of dyspnea were correlated with exertional exhaustion (CFS criteria), poor Physical Functioning (SF-36), McGill Pain Scores, and the severity of rapid heart rate, muscle spasms, and dizziness. CFS and control subjects had normal pulmonary function indicating the absence of vocal cord dysfunction, asthma, or COPD. However, the CFS group had higher sensory and perceptual scores for dyspnea following spirometry. Sensory hypersensitivity without any airflow obstruction may be responsible for the subjective perception of dyspnea in CFS. Although the CFS subjects had higher depression and anxiety questionnaire scores, these states did not correlate with their complaints of dyspnea. CFS should be considered in dyspneic subjects who have complex fatigue, pain, and multisystem complaints.

### Author Contributions

JNB was the Principal Investigator of the studies, and supervised the clinical studies performed by MKR, YZ, ULH, CRT, SM and RE. CR introduced the MRC Dyspnea Scores in the initial studies. MC and MRK performed the pulmonary function testing. JNB and MKR prepared the manuscript with OA conducting the statistical analysis. All authors have read and approved this manuscript.
